# Cannabis body packing: a case report

**DOI:** 10.11604/pamj.2016.24.327.9450

**Published:** 2016-08-30

**Authors:** William Ngatchou, Daniel Lemogoum, Basile Essola, Ahmed Ramadan, Michèle Ngassa, Virginie Guimfacq, Pierre Mols, Pierre Youatou Towo

**Affiliations:** 1 Department of Cardiac Surgery, St Pierre Hospital, Brussels, Belgium; 2 Department of Emergency Medicine, St Pierre Hospital, Brussels, Belgium; 3 Medicine Faculty, Douala University, Cameroon; 4 Department of Gastroenterology, Brugmann Hospital, Brussels, Belgium; 5 Department of Cardiology, Ixelles Hospital Brussels, Brussels, Belgium

**Keywords:** Cannabis, bodypacking, mule

## Abstract

Drug traffic is a major concern worldwide. We report a case of a 27-year old male who presented with a diffuse abdominal plain to the emergency department. Abdominal X-ray demonstrated multiple foreign bodies along the intestinal tract, which were found to be cannabis packets. The patient was treated conservatively with a good result.

## Introduction

Due to the constant need for rapid financial gain, the international drug trade remains a major concern worldwide [1, 2]. First reported in 1973, Body packing describes the intracorporeal concealment of illicit substance in the alimentary tract [3]. "Body packers" also known as "drug mules", "swallowers", "internal carriers" or "couriers" usually carry about one Kg of drug divided into 50-100 packets of 8-10 g each. A wide range of illicit drugs may be transported in this way, including cocaine, heroin, hashish, amphetamines and "ectasy" [1, 2]. Emergency department physicians must recognize and look for the signs of complications that these drug packages can cause. These include small bowel or large bowel occlusion, gastro intestinal perforation. In addition, package rupture can cause systemic drug absorption, resulting in drug toxicity and death [1–4]. Asymptomatic patient must be treated conservatively with or without laxative [1–3]. The need of surgical intervention is currently rare. The main indications for emergency surgical intervention are suspected packet rupture, bowel obstruction or prolonged packet retention [1–4].

## Patient and observation

**Figure 1:**
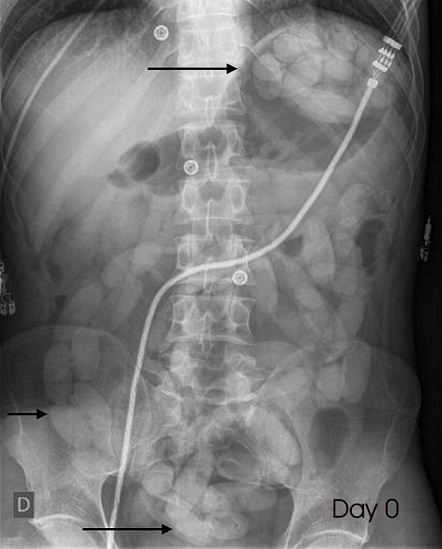
Plain abdominal X-ray at admission with multiple body packers (arrows)

**Figure 2:**
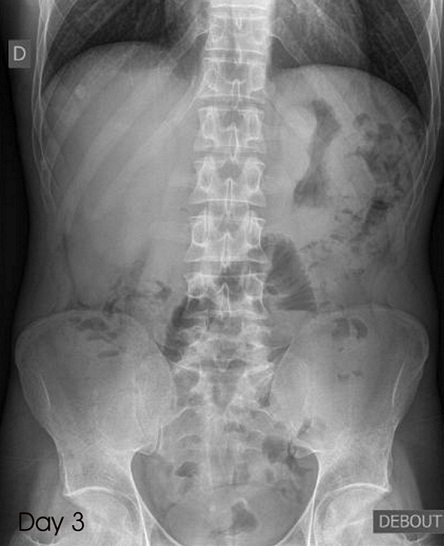
Plain abdominal X-ray after 3 days

A 27-year old male presented to our emergency ward with a complaint of diffuse abdominal pain started 6 hours before. He was afraid because the day before he has ingested 4 kilogram of hashish. Physical examinations reveal a cardiac rhythm of 92 bpm, a blood pressure of 132/ 78 mmHg and diffuse abdominal tenderness without defense. Blood tests and urine drug screening were normal. A plain abdominal X-ray demonstrated multiple "body packers" in the stomach, along the intestinal tract and the rectum (Figure 1). We started oral and rectal laxative treatment and the patient was admitted in intensive care unit. After three days, the patient eliminated spontaneously all the packets and was discharged from hospital (Figure 2).

## Discussion

Our case emphasizes the safety of conservative management of Body packing syndrome. Ingestion of illicit drug packages has increased worldwide and become a growing challenge for a wide range of specialist including emergency physicians, internist, radiologist, surgeons and toxicologists [1–4]. The first step is to confirm the suspicion by anamnesis and physical examination, which is not easy because drug dwellers usually would not coopered. Plain abdominal X-ray is the radiological examination use to detect packets and to confirm that all packages have been removed with a sensitivity from 85to 90% [5]. In case of negative abdominal plain films with remain strongly suspicion of body packing or retained packets, low dose abdominal CT scan can be use [1, 2, 5]. On plain abdominal radiographs, the radiologist should search for the presence of following findings: one or multiple well-defined opacities in the stomach, small bowel or large bowel that are not suggestive of alimentary content, the "double condom sign", defined as a clear crescent of air bordering an ovoid opacity; a smooth and uniformly shaped oblong structure "tic-tac sign"; the "parallelism sign" defined as firm packages aligning parallel to each other in the bowel lumen [1, 2, 4]. Depend for the drug ingested various clinical symptoms mays occur. Cocaine may cause agitation, tachycardia, hypertension, sweating, dilated pupils and hyperthermia [1, 2, 4]. More serious effects are status epilepticus, seizures, myocardial infarction and ventricular fibrillation. Heroin may produce a decrease level of consciousness, respiratory depression and pinpoint pupils [2]. Hashish toxicity may result in tachycardia, postural hypotension, conjunctival injection and ataxia. Psychiatric reaction including euphoria, anxiety, time-space distortions, fear, distrust, dysphoria, or panic disorder. Visual hallucinations and acute paranoid psychosis may occur with high doses [6]. Early surgical removal of the packets is recommended in patient who present with toxicity sign or mechanical gastrointestinal obstruction [1, 2, 7]. Specific antidote therapy should also be administrated whenever it is available [1, 2]. Fortunately, with the improvement of wrapping technique, the percentage of intoxication may actually be decreasing and less than 5% of body packers will undergo surgical treatment [1–7]. Asymptomatic body packers should be monitoring closely, preferably on an intermediate or intensive care unit, allowing for a quick response in case of complications or clinical detoriation [8]. Elimination of packets in asymptomatic patient can be passive by normal bowel movements [5] or active by using laxatives and bowel irrigation [1, 2]. Oiled laxatives and endoscopic removal of packets should not be applied due to high risk of perforating the latex wrapping [8]. The average length of hospital stay range from 2.8days in conservative to 10.4 days for surgical management of body packers [8].

## Conclusion

Conservative management of body packers is safe. Cases suspected of poisoning or gastrointestinal obstruction should undergo emergency surgical intervention.
